# Novel Inhibitors of Cholesterol Degradation in *Mycobacterium tuberculosis* Reveal How the Bacterium’s Metabolism Is Constrained by the Intracellular Environment

**DOI:** 10.1371/journal.ppat.1004679

**Published:** 2015-02-12

**Authors:** Brian C. VanderVen, Ruth J. Fahey, Wonsik Lee, Yancheng Liu, Robert B. Abramovitch, Christine Memmott, Adam M. Crowe, Lindsay D. Eltis, Emanuele Perola, David D. Deininger, Tiansheng Wang, Christopher P. Locher, David G. Russell

**Affiliations:** 1 Department of Microbiology and Immunology, Cornell University, Ithaca, New York, United States of America; 2 Vertex Pharmaceuticals Incorporated, Boston, Massachusetts, United States of America; 3 Departments of Microbiology and Biochemistry, University of British Columbia, Vancouver, British Columbia, Canada; National Institutes of Health, UNITED STATES

## Abstract

*Mycobacterium tuberculosis* (Mtb) relies on a specialized set of metabolic pathways to support growth in macrophages. By conducting an extensive, unbiased chemical screen to identify small molecules that inhibit Mtb metabolism within macrophages, we identified a significant number of novel compounds that limit Mtb growth in macrophages and in medium containing cholesterol as the principle carbon source. Based on this observation, we developed a chemical-rescue strategy to identify compounds that target metabolic enzymes involved in cholesterol metabolism. This approach identified two compounds that inhibit the HsaAB enzyme complex, which is required for complete degradation of the cholesterol A/B rings. The strategy also identified an inhibitor of PrpC, the 2-methylcitrate synthase, which is required for assimilation of cholesterol-derived propionyl-CoA into the TCA cycle. These chemical probes represent new classes of inhibitors with novel modes of action, and target metabolic pathways required to support growth of Mtb in its host cell. The screen also revealed a structurally-diverse set of compounds that target additional stage(s) of cholesterol utilization. Mutants resistant to this class of compounds are defective in the bacterial adenylate cyclase Rv1625/Cya. These data implicate cyclic-AMP (cAMP) in regulating cholesterol utilization in Mtb, and are consistent with published reports indicating that propionate metabolism is regulated by cAMP levels. Intriguingly, reversal of the cholesterol-dependent growth inhibition caused by this subset of compounds could be achieved by supplementing the media with acetate, but not with glucose, indicating that Mtb is subject to a unique form of metabolic constraint induced by the presence of cholesterol.

## Author Summary

Human beings are the sole ecological niche for *M*. *tuberculosis* (Mtb), and it is estimated that 1.8 billion people are currently infected with Mtb. An important aspect of this infection is Mtb’s ability to maintain infection by replicating within macrophages. Within macrophages, Mtb exploits a specialized set of metabolic pathways to utilize host-derived nutrients, such as fatty acids and/or cholesterol, for energy production. Many details regarding Mtb metabolism during infection remain unknown. Here we took a chemical approach to identify small molecule probes, which target Mtb metabolism during infection in macrophages. We found that many of the small molecule inhibitors that we identified require cholesterol for activity. Here we report a novel chemical rescue approach to identify the metabolic targets of three novel inhibitors, and discovered that cAMP signaling is linked to cholesterol utilization in Mtb. Together, these data demonstrate that cholesterol exerts a dominant effect on *Mtb* metabolism within macrophages. Additionally, the novel inhibitors identified in this study will facilitate evaluation of cholesterol metabolism as a target for chemotherapeutic intervention.

## Introduction

There is an urgent need to identify new drugs to treat *Mycobacterium tuberculosis* (Mtb). The World Health Organization estimates that 1.8 billion people are infected with *Mycobacterium tuberculosis* (Mtb) and approximately 1.3 million people die from tuberculosis (TB) annually. The global prevalence of TB is sustained by the ongoing HIV-AIDS pandemic, poverty, and the emergence of antibiotic resistant isolates of Mtb [1]. Unfortunately, with the notable exception of bedaquiline [2], there have been no new drugs approved for treatment of tuberculosis, and some of the emergent drug resistant strains are virtually untreatable. Therefore identification of compounds that inhibit new biological targets and pathways is a vital component in TB drug discovery.

Intracellular survival within macrophages is an important aspect of Mtb pathogenesis. In macrophages Mtb resides and replicates primarily in phagosomes, which are thought to be a nutritionally-constrained environment [3,4]. In order to replicate in this environment Mtb relies on particular metabolic pathways to utilize host-derived nutrients [5]. Numerous transcriptional profiling studies have indicated that the metabolism of host-derived carbon sources such as fatty acids and/or cholesterol are critical for Mtb survival in macrophages [6–10]. Additionally, genetic studies have identified key bottlenecks in Mtb carbon metabolism, which are essential for growth during infection. Specifically, mutants lacking genes involved in gluconeogenesis [11–13], cholesterol utilization [14–17], or the methyl citrate cycle (MCC) [18,19] fail to establish infection in macrophages. The importance of these pathways is underscored by the observation that many of these pathways are also required for full Mtb pathogenicity in small animal models of infection. For this reason, the central carbon metabolic pathways of Mtb are considered potential targets for TB drug discovery.

Identifying small molecules that inhibit predetermined enzymatic targets in Mtb with target-based screens continues to be a challenge. Frequently inhibitors identified through target-based screens fail to show activity when tested against intact, live Mtb. Such failures are usually the consequence of poor permeability, drug efflux, and/or metabolic redundancy [20]. In contrast, cell-based screens typically identify compounds on the basis of their activity against live Mtb but this approach is constrained both by appropriateness of the growth condition(s) used in the screen, and the subsequent need to determine the mode of action of inhibitors. To circumvent at least some of these challenges, phenotypic screening against Mtb-infected macrophages represents a viable alternative strategy.

In this report we conducted an unbiased chemical screen to identify compounds that inhibit Mtb replication during infection in macrophages, and subsequently in cholesterol media. To isolate compounds that specifically target cholesterol metabolism in Mtb we developed a novel chemical-rescue approach that exploits the toxicity of cholesterol-derived intermediates to identify pathway specific inhibitors in whole Mtb. With this approach we identified inhibitors of the HsaAB complex, which is required for complete degradation of the A/B rings of cholesterol, and PrpC, the gating enzyme of the MCC, which is required for effective assimilation of propionyl-CoA into central metabolism. Finally, we describe three structurally-diverse compounds that limit cholesterol utilization indirectly by perturbing cyclic-AMP (cAMP) levels. The sheer breadth of inhibitory compounds that reduce Mtb fitness in macrophages through the disruption of cholesterol metabolism was unexpected but provides us with a rich set of new tools to probe the metabolic re-alignment required to sustain growth within the host macrophage.

## Results

### Identification of compounds that inhibit Mtb growth in macrophages

To discover compounds that limit Mtb growth within macrophages, we developed a whole-cell assay suitable for phenotypic high-throughput screening (HTS). In a 384-well format, J774 macrophages were infected with an Mtb strain that constitutively expresses the fluorescent protein mCherry. In this assay, Mtb replicates and produces a 4- to 5-fold increase in mCherry signal over a six-day period. In the presence of the frontline anti-TB drug rifampicin, the Mtb mCherry signal is quenched in a concentration-dependent manner indicating that mCherry fluorescence can serve as a marker of reduced intracellular Mtb growth (S1 Fig.). To identify compounds that inhibit Mtb growth in macrophages, the J774 cells were infected and screened with an experimental compound library at a single concentration of 10 μM. We quantified the Mtb derived-mCherry signal at day 6 and hit compounds were identified by their ability to reduce Mtb-derived mCherry fluorescence. For the screen we used a proprietary compound library supplied by Vertex Pharmaceuticals which contained ~340,000 synthetic small molecules and natural products. From this screen we identified ~4000 compounds that displayed Mtb growth inhibition in the range of 30–100% relative to the positive control rifampin (5 μM) which we used as the reference for 100% inhibition. The cutoff of 30% inhibition is a low stringency filter and was chosen because this threshold is approximately 3 standard deviations from the mean signals from the experimental compounds in our screen (S1 Fig.). The calculated Z’-factors for all of the ~1,200 screening assay plates was 0.65–0.75, indicating that the assay is very robust [21]. We next determined the potency of the hit compounds by testing a compound dilution series against Mtb using the intracellular mCherry fluorescence assay. This assay reconfirmed activity for >90% of the hit compounds and resulted in 1,359 validated hits with IC_50_ values <50.0 μM in the macrophage assay.

**Fig 1:**
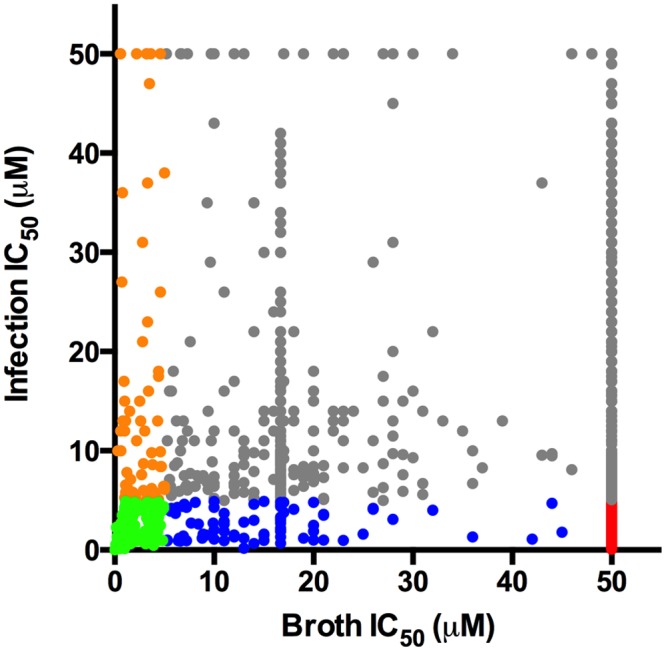
Distribution of hit compound IC_50_ values in macrophages and in 7H9 OADC. Dot plot depicting the IC_50_ values for the most potent 1,359 compounds in 7H9 OADC and in the macrophage infection assays. For both assays, compounds were tested across 8 separate 2-fold dilution series 50–0.4 μM. Universally active compounds with IC_50_ values < 5.0 μM in macrophages and < 5.0 μM in 7H9 OADC are indicated in green. The conditionally active compounds with IC_50_ values < 5.0 μM in macrophages and > 50.0 μM 7H9 OADC are depicted in red. Compounds with differential inhibitory activity are indicated in orange, grey, and blue.

Middlebrook 7H9 OADC media has historically been used to evaluate anti-Mtb compounds. Therefore, we titrated our most potent 1,359 hits against Mtb cultivated in standard 7H9 OADC and quantified growth inhibition using an Alamar Blue-based assay [22]. This revealed two distinct sub-sets of compounds; those that were universally-active, inhibiting Mtb growth in 7H9 OADC and inside macrophages, and those that were conditionally-active, that inhibit intracellular Mtb growth but have little or no inhibitory activity in 7H9 OADC. Of the 1,359 hits tested in this assay, 141 (10%) were universally-active compounds with IC_50_ values <5.0 μM in 7H9 OADC media and in the macrophage assay (Fig. 1). The screening library that we used contained known anti-Mtb compounds and greater than 70% of the universally active compounds were structurally related to compounds with reported anti-Mtb activity. The most potent conditionally-active subset contained 132 (9%) compounds that inhibit Mtb replication in macrophages displaying IC_50_ values <5.0 μM. These conditionally-active compounds demonstrate little inhibitory activity against Mtb in 7H9 OADC media (IC_50_ values >50.0 μM). Importantly >95% the compounds in the conditionally-active category were novel with no structurally related compounds reported in the literature. Numerous compounds displayed differential potency in these two different assays which may be a result from multiple factors including: (*i*) variable compound access to Mtb, (*ii*), induction of bacterial drug efflux systems during infection, (*iii*) partial inactivation of the compound by host-cell metabolism, or (*iv*) the compounds target pathways (host or bacterial) required only during infection.

### Numerous conditionally-active compounds are active in medium containing cholesterol

Middlebrook 7H9 OADC media is a carbohydrate-rich medium that does not reflect the nutritional conditions encountered by Mtb in macrophages, or any aspect of the bacterium’s life cycle. We hypothesized that a subset of the conditionally active compounds target Mtb metabolism and inhibition by these compounds would be buffered by the nutritional redundancy provided within 7H9 OADC medium. Numerous studies have indicated that host-derived lipid (cholesterol and fatty acid) substrates and the metabolic pathways required for their utilization are important for Mtb replication during infection [11,16,23,24]. Therefore, we tested the 132 most potent conditionally-active compounds (IC_50_ values < 5.0 μM in macrophages) against Mtb grown in 7H12 media containing cholesterol as the main carbon source [25]. We observed that 74 (56%) of these conditionally-active compounds inhibited Mtb replication in this medium with IC_50_ values <5.0 μM. These IC_50_ values are comparable to those observed in the macrophage assay. Additionally, 33 of these 74 compounds were also active in 7H12 media containing acetate as the carbon source with IC_50_ values < 5.0 μM. Of the 132 conditionally-active compounds, we were unable to recover inhibitory activity for 58 conditional hits despite testing various liquid culture conditions. Possible explanations for this include: (*i*) our inability to faithfully reproduce the environment of the macrophage in liquid media; (*ii*) the compounds target the host-cell; (*iii*) the compounds are pro-drugs that require activation by an unknown enzyme (host or bacterial); or (*iv*) the compounds limit bacterial growth by inducing macrophage cell death. Nonetheless, we isolated numerous potent compounds that inhibit Mtb growth in cholesterol media and we hypothesize that a subset of these compounds target Mtb metabolic pathways involved in cholesterol utilization.

### Chemical-rescue of cholesterol toxicity in Δicl1 Mtb

In Mtb, the enzyme isocitrate lyase (Icl1) is bifunctional, acting both as an isocitrate lyase in the glyoxylate pathway and as a methyl-isocitrate lyase of the methylcitrate cycle (MCC) [26]. Mtb utilizes the MCC to assimilate propionyl-CoA into central metabolism to produce succinate and pyruvate [19,27]. When an Icl1 deficient strain (Δicl1 Mtb) is growth in 7H9 OADC supplemented with cholesterol or propionate this mutant experiences a metabolic toxicity and fails to grow despite the presence of saturating amounts of carbohydrates and fatty acids in the medium. We hypothesize that this toxicity is induced by intermediates of the MCC that accumulate in Δicl1 Mtb when the bacteria are supplied either cholesterol or propionate. We further hypothesized that chemical inactivation of key cholesterol catabolic enzymes and/or MCC enzymes will alleviate this toxicity and rescue growth inhibition in the Δicl1 Mtb mutant grown in 7H9 OADC supplemented with cholesterol.

**Fig 2:**
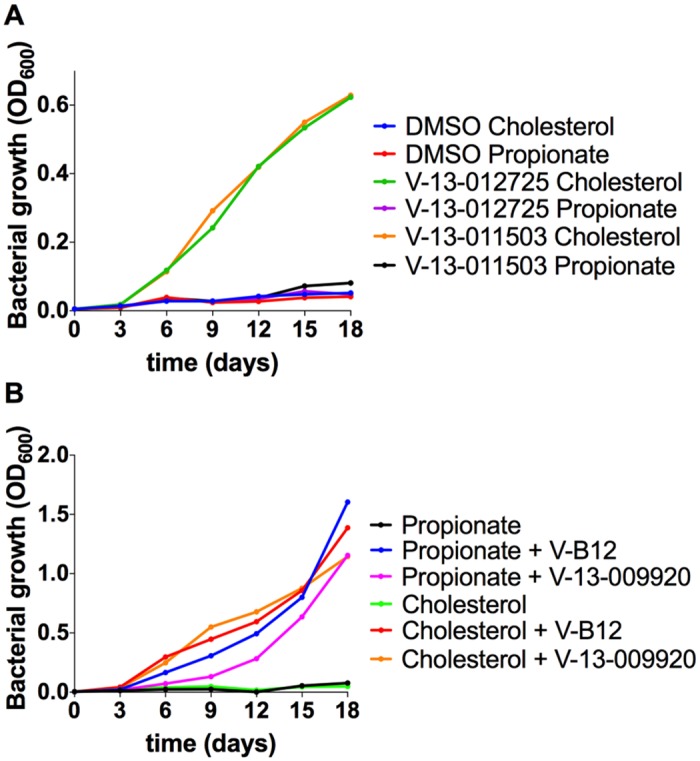
Chemical rescue of Mtb ΔIcl1. **(A)** Growth of Mtb ΔIcl1 was monitored in 7H9 OADC containing cholesterol (100 μM) or propionate (100 μM) in the presence of V-13–012725 (25 μM) and V-13–011503 (25 μM). Growth rescue by the compounds V-13–012725 and V-13–011503 is specific to cholesterol with no growth is observed in media containing propionate. **(B)** The compound, V-13–009920 (25 μM) rescues Mtb ΔIcl1 growth in 7H9 OADC media containing cholesterol (100 μM) and propionate (100 μM). Chemical rescue by V-13–009920 is comparable to rescue by vitamin-B12 (10 μg/ml). The data are representative of two independent experiments.

To identify compounds that suppress cholesterol toxicity in Δicl1 Mtb we evaluated our most potent 1,359 hit compounds for their ability to rescue cholesterol-dependent toxicity in Δicl1 Mtb. Briefly, a Δicl1 Mtb strain, which constitutively expresses mCherry, was inoculated into Middlebrook 7H9 OADC media containing 100 μM cholesterol and compounds at 10 μM. Bacterial growth was measured by quantifying the bacterial-derived mCherry fluorescence at day 12. From this single-point analysis we identified three compounds (V-13–009920, V-13–012725, and V-13–011503) that restored Δicl1 Mtb growth in the presence of cholesterol suggesting that these compounds target key enzymes of the MCC or the cholesterol breakdown pathway (S2 Fig.). To discriminate between compounds that potentially target the MCC enzymes from those that target cholesterol catabolism, we also evaluated these compounds for their ability to rescue growth in the presence of propionate. While all three compounds restored growth of the Δicl1 Mtb in 7H9 OADC supplemented with cholesterol, only one compound, V-13–009920, restored bacterial growth on propionate (S4 Fig.). We next quantified the relative growth rates of Δicl1 Mtb in 7H9 OADC supplemented with cholesterol or propionate in the presence of these compounds. This confirmed that V-13–012725, and V-13–011503 rescue growth of Δicl1 Mtb only in the presence of cholesterol while V-13–009920 rescues growth of Δicl1 Mtb in the presence of cholesterol and propionate (Fig. 2). Additionally, the growth rescue of Δicl1 Mtb by V-13–009920 in 7H9 OADC is equivalent to rerouting propionyl-CoA into the methyl-malonyl pathway upon the addition of vitamin B12 [28] Based on these phenotypes, we hypothesized that V-13–009920 targets enzymes of the MCC while V-13–011503 and V-13–012725 target cholesterol catabolic enzymes upstream of the MCC.

### V-13–009920 inhibits the 2-methylcitrate synthase PrpC

**Fig 3:**
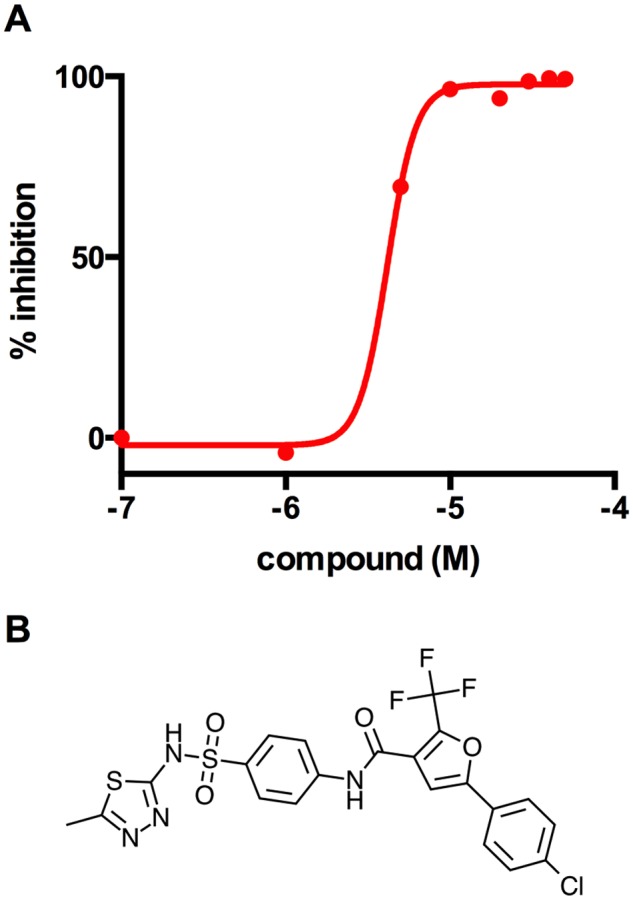
V-13–009920 inhibits the 2-methylcitrate synthase PrpC. **(A)** Inhibition of PrpC enzyme activity was monitored by quantifying thiol release from propionyl-CoA leading to the formation of 3-thio-6-nitrobenzoate from DTNB measured at 412 nm. V-13–009920 inhibits pure PrpC enzyme with an IC_50_ value of 4.0 ± 1.1 uM. (**B**) Chemical structure of V-13–009920 (5-(4-chlorophenyl)-*N*-(4-(*N*-(5-methyl-1,3,4-thiadiazol-2-yl)sulfamoyl)phenyl)-2-(trifluoromethyl)furan-3-carboxamide. Data are representative of two independent experiments.

Since V-13–009920 rescues Δicl1 Mtb growth in propionate, the target of this compound is most likely an enzyme in the MCC. PrpC is the 2-methylcitrate synthase that catalyzes the first dedicated reaction of the MCC in Mtb and condenses oxaloacetate with propionyl-CoA to form 2-methylcitrate [19]. We therefore tested V-13–009920 against recombinant PrpC and confirmed that V-13–009920 directly inhibits pure PrpC enzyme activity *in vitro* with an IC_50_ of 4.0 ± 1.1 μM (Fig. 3). The compound V-13–009920 has an IC_50_ value of 3.0 μM in macrophages and is 10-fold more potent in 7H12 cholesterol media with an IC_50_ value of 0.3 μM. Microbiological profiling experiments demonstrate that this compound is bacteriostatic against Mtb grown in 7H12 cholesterol media. Previous reports have established that a *prpCD* double mutant has a growth defect in macrophages [19]. Thus, our discovery of a PrpC inhibitor from a collection of small molecules that limit intracellular Mtb replication reassured us that we have identified compounds that target the Mtb metabolic pathways required for infection.

The ability of the PrpC inhibitor to promote growth of Δicl1 Mtb in 7H9 OADC media containing cholesterol is consistent with our interpretation that a toxic MCC intermediate(s) is produced in Δicl1 Mtb under this condition. Eoh and Rhee recently reported that the growth defect observed in Δicl1 Mtb during growth solely on propionate is derived from a defective MCC and is principally due to a depletion of tricarboxylic acid (TCA) intermediates and the secondary accumulation of potentially toxic intermediates. Additionally, this study reported that vitamin-B12 rescues the Δicl1 Mtb growth defect by shunting carbons from propionyl-CoA back into the TCA cycle via the vitamin-B12 dependent methyl-malonyl pathway [28]. Under the conditions used here it is unlikely that TCA intermediate levels are limiting in 7H9 OADC cholesterol, which contains excess amounts of glucose, fatty acids, and glycerol. Additionally, unlike the vitamin-B12 rescue of Δicl1 Mtb, chemical inactivation of PrpC would not reroute carbons into the TCA cycle. Thus, we propose that growth suppression of Δicl1 Mtb under this condition is induced by accumulation of a toxic intermediate(s) produced from the MCC.

### V-13–011503 and V-13–012725 inhibit the two-component flavin-dependent hydroxylase HsaAB

**Fig 4:**
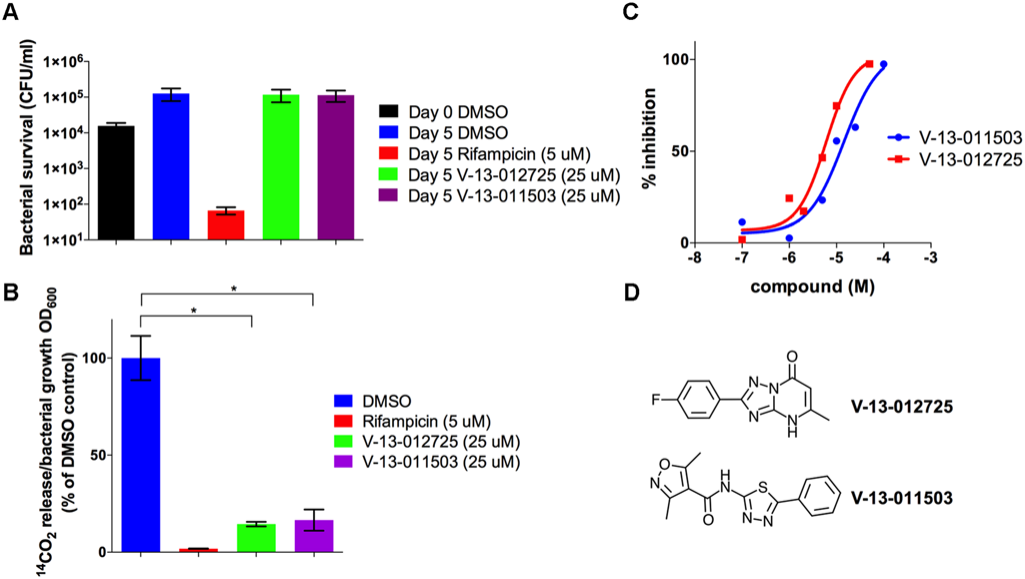
V-13–012725 and V-13–011503 inhibit cholesterol breakdown. **(A)** Growth of wild type Mtb is not inhibited in 7H9 OADC containing cholesterol (100 μM) and experimental compounds. **(B)** In 7H9 OADC containing cholesterol (100 μM) V-13–012725 and V-13–011503 specifically inhibit cholesterol turnover. **(C)** V-13–012725 and V-13–011503 directly inhibit the activity of the recombinant HsaAB enzyme complex with IC_50_ values of 5.0 ± 0.8 and 11.0 ± 2.0 μM, respectively. **(D)** Chemical structures of V-13–012725, 2-(4-fluorophenyl)-5-methyl-1H-[1, 2, 4]triazolo[1, 5-a]pyrimidin-7-one and V-13–011503, 3,5-dimethyl-N-(5-phenyl-1,3,4-thiadiazol-2-yl)-1,2-oxazole-4-carboxamide. Data are representative of at least two independent experiments and error bars represent s.d.

To determine if the compounds V-13–011503 and V-13–012725 directly inhibit cholesterol catabolism, we monitored the evolution of ^14^CO_2_ from [4–^14^C]-cholesterol by radiorespirometry. For this, wild-type Mtb was grown in 7H9 OADC supplemented with 100 μM cholesterol and trace levels of [4–^14^C]-cholesterol. In this assay the bacteria are provided excess carbohydrates and fatty acids to support bacterial growth allowing us to specifically quantify inhibition of cholesterol catabolism and bacterial viability in this assay was confirmed (Fig. 4A). Under this condition we observed that both V-13–012725 and V-13–011503 significantly decreased the levels of ^14^CO_2_ released in the presence of these compounds (Fig. 4B).

To delineate the targets of these inhibitors we next analyzed the cholesterol-derived metabolites which accumulate in Mtb following treatment with V-13–012725 and V-13–011503. GC-MS analyses of culture extracts from cells treated with V-13–012725 and V-13–011503 revealed one diagnostic metabolite (*t*_R_ = 14.9 min) that was undetectable in DMSO-treated cells (S3 Fig.). The retention time and mass spectrum of this metabolite corresponded to 3-hydroxy-9,10-seconandrost-1,3,5(10)-triene-9,17-dione (3-HSA) [29]. Treatment with V-13–012725 also promoted the accumulation of two additional metabolites and the retention times and mass spectrum of these metabolites correspond to those of 3-hydroxy-9-oxo-9,10-seco-23,24-bisnorchola-1,3,5(10)-trien-22-oic acid (3-HSBNC) [30] and a derivative of 3-HSBNC with a double bond. Most importantly, accumulation of 3-HSA following treatment with V-13–011503 and V-13–012725 indicates that these compounds target enzymes involved in degrading the A/B rings of cholesterol in Mtb (S3 Fig.).

To identify the molecular targets of V-13–011503 and V-13–012725 we tested these two compounds for their ability to inhibit key enzymes involved in the degradation of the A/B rings of cholesterol (HsaA, HsaB, HsaC, or HsaD). At concentrations up to 100 μM, neither V-13–011503 nor V-13–012725 detectably inhibited HsaC or HsaD in *in vitro* enzymatic assays described previously [31,32]. However, both compounds inhibited HsaAB, the two-component flavin-dependent hydroxylase that catalyzes the 4-hydroxylation of ring A to produce a catechol. HsaAB inhibition was measured using a coupled enzymatic reaction containing recombinant HsaAB, HsaC, and 3-HSA which allowed us to track 4,9-DHSA production by measuring absorbance at 392 nm as described in the methods. Dose-response assays with these compounds revealed that the IC_50_ values for the inhibitors V-13–011503 and V-13–012725 are 11 ± 2 μM and 5.0 ± 0.8 μM, respectively against pure enzymes (Fig. 4C-D). Killing kinetic analysis revealed that these two compounds are bacteriostatic against Mtb in media containing cholesterol as a sole carbon source. Our metabolite analysis confirmed that the side chain of cholesterol is fully degraded to 3-HSA upon treatment with the HsaAB inhibitors therefore, it is likely that Mtb can support minimal growth on cholesterol as a sole carbon source *in vitro* by utilizing the carbons liberated from the side chain of cholesterol in the presence of these inhibitors.

### Characterizing the Mtb response to the orphan cholesterol utilization inhibitors

A large proportion of hit compounds were active against Mtb when the bacterium is grown in cholesterol medium but do not seem to target cholesterol catabolism directly. To further characterize this diverse collection of compounds we performed global gene expression profiling to identify conserved patterns of differentially expressed genes to provide indication of mode of action [33,34]. Using this approach, we focused on three structurally-diverse compounds from the 132 conditionally-active inhibitors that are among the most potent in macrophages and cholesterol media (S4 Fig.). Briefly, Mtb was cultivated in 7H12 cholesterol media and exposed to compounds at 10x IC_50_ concentration for 4 hr and the global Mtb transcriptional responses were quantified by microarray. We focused on those genes which were significantly up- or down-regulated across 3 biological replicates [8].

**Fig 5:**
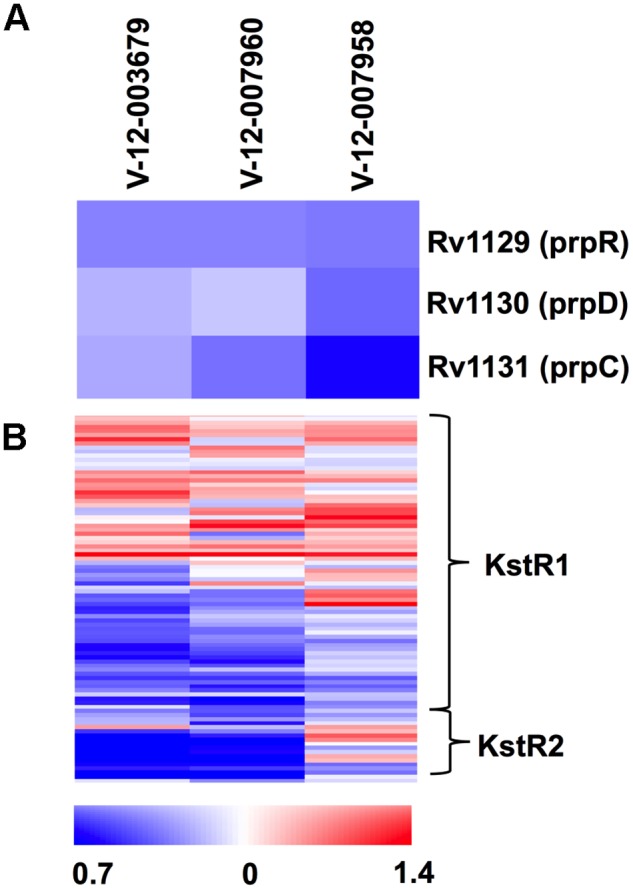
Orphan cholesterol utilization inhibitors display transcriptional response characteristic of blocked cholesterol utilization. **(A)** The methylcitrate genes are repressed in the presence of the Group 1 cholesterol utilization inhibitors. **(B)** Numerous genes in the KstR1 and KstR2 regulons are repressed in the presence of the orphan cholesterol utilization inhibitors. Data represent the normalized mean of three independent experiments with P-values <0.05. Data represents the ratio of gene expression values compared to DMSO controls.

These inhibitors induced a common set of 49 genes including genes associated with the putative drug efflux systems *Rv1216–19c* and *Rv0677–78c* (S1 Table). *Rv1216–19c* encodes a putative ABC-type transporter (*Rv1218c*), which has been implicated in the efflux of a wide-variety of small molecule substrates from *Mtb* [35]. Similarly, we noted up-regulation of the genes *Rv0676–78c*, which encode the putative MmpL5 efflux pump that has been implicated in azole drug, clofazimine, and bedaquiline resistance [36,37]. Importantly, these structurally-unrelated compounds shared a common transcriptional profile consistent with a perturbation in cholesterol utilization. The key feature of this transcriptional signature is the repression of the MCC genes despite the presence of cholesterol in the medium (Fig. 5). This is informative since the MCC is involved in assimilating cholesterol-derived propionyl-CoA and the expression of the MCC genes are normally highly induced in the presence of increasing concentrations of propionate and/or cholesterol [27,38]. Additionally, 21 common genes that are under control of the transcriptional regulators KstR1 and KstR2 are also repressed and these genes are also normally induced during infection or in the presence of cholesterol or cholesterol breakdown products (Fig. 5 and S1 Table) [39,40]. The concomitant repression of the MCC genes and genes within KstR1 and KstR2 regulons suggest that cholesterol utilization is blocked in the presence of these inhibitors. Growth arrest was also evident in the transcriptional response to these compounds as indicated by the reduced expression of ribosome-encoding genes (S1 Table). The overlapping gene lists from these transcriptional responses is an unexpected result given the structural diversity of these compounds and we hypothesize that these compounds target aspect(s) early in cholesterol utilization.

### Phenotypic characterization of orphan cholesterol utilization inhibitors

**Fig 6:**
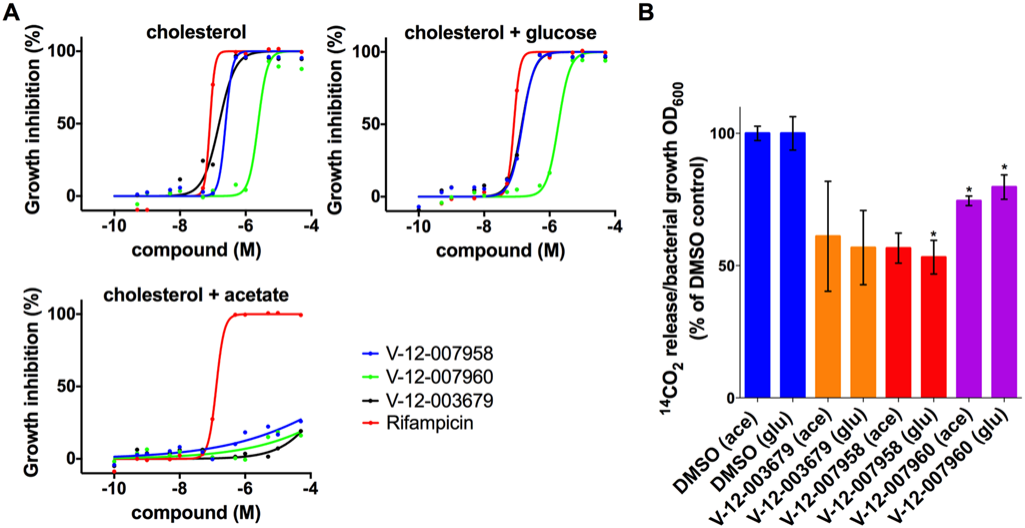
Acetate rescues inhibition by orphan cholesterol utilization inhibitors. **(A)** Wild type Mtb was grown in 7H12 media containing cholesterol (100 μM) and supplemented with the additional carbon substrates glucose (0.1%) and acetate (0.1%) as indicated. Growth inhibition was quantified using an Alamar Blue-based assay. **(B)** The compounds V-12–003679, V-12–007958, and V-12–007960 inhibit cholesterol utilization in media supplemented with cholesterol and the additional carbon substrates 0.1% glucose (glu) and 0.1% acetate (ace) indicated in parentheses. Data are representative of at least two independent experiments and error bars represent s.d.

Although lipids appear to be favored nutrients by Mtb during infection, the bacterium likely encounters complex mixtures of nutrients *in vivo*. We next examined the inhibitory activities of these orphan cholesterol utilization inhibitors in the presence of multiple carbon sources. This analysis revealed that in mixed carbon source media, containing both cholesterol and a glycolytic substrate (glucose), these compounds inhibit Mtb replication with potencies similar to the intracellular macrophage assay. However, in mixed carbon source media containing cholesterol and a gluconeogenic substrate (acetate), these compounds do not inhibit Mtb replication (Fig. 6A). Previous metabolomics analysis has shown that Mtb can co-metabolize simple carbon substrates such as glucose, glycerol and acetate, although the products of these substrates have separate fates [41]. Our observation suggests that these compounds inhibit Mtb growth by limiting cholesterol turnover and acetate likely rescues this defect by fueling the TCA cycle for energy production. To test this idea we quantified cholesterol utilization by Mtb in the same mixed carbon source media by monitoring the release of ^14^C-labeled CO_2_ from [4–^14^C]-cholesterol by radiorespirometry. Consistent with our inhibition observations these compounds inhibited cholesterol utilization equally in mixed carbon source media containing either a glycolytic or gluconeogenic substrate (Fig. 6B). Importantly, because acetate rescues growth inhibition the reduction in the amount of ^14^CO_2_ release by these compounds is not linked to a reduction in bacterial growth when the media is supplemented with acetate. Analysis of the killing kinetics with this set of inhibitors indicates that these compounds are bacteriostatic against Mtb in both macrophages and in cholesterol media.

### A role for cAMP in regulating cholesterol utilization in Mtb

In comparison to the HsaAB inhibitors, the orphan cholesterol inhibitors (V-12–003679, V-12–007958, and V-12–007960) only partly inhibit cholesterol metabolism leading to a 30–50% reduction in cholesterol turnover. In addition, these inhibitors do not rescue growth of Δicl1 Mtb in 7H9 OADC supplemented with cholesterol and we could not detect accumulation of any cholesterol-derived intermediate in the presence of these compounds. These observations lead us to hypothesize that these inhibitors could indirectly inhibit cholesterol utilization in Mtb by perturbing a regulatory system that modulates cholesterol or propionyl-CoA utilization. To identify mutants resistant to growth inhibition by these compounds we grew a transposon library (~10^5^) wild type CDC1551 background, in 7H12 cholesterol media containing V-12–007958 (10 μM) for seven days and plated the mutant pool onto 7H10 agar plates without compound and cholesterol. We picked 10 mutants and, upon sequencing, we identified 5 insertions that mapped to the adenylate cyclase *rv1625c*/*cya* (three independent insertion sites). The integral membrane adenylate cyclase Cya is known to generate cyclic adenosine 3′,5′-monophosphate from ATP [42,43].

**Fig 7:**
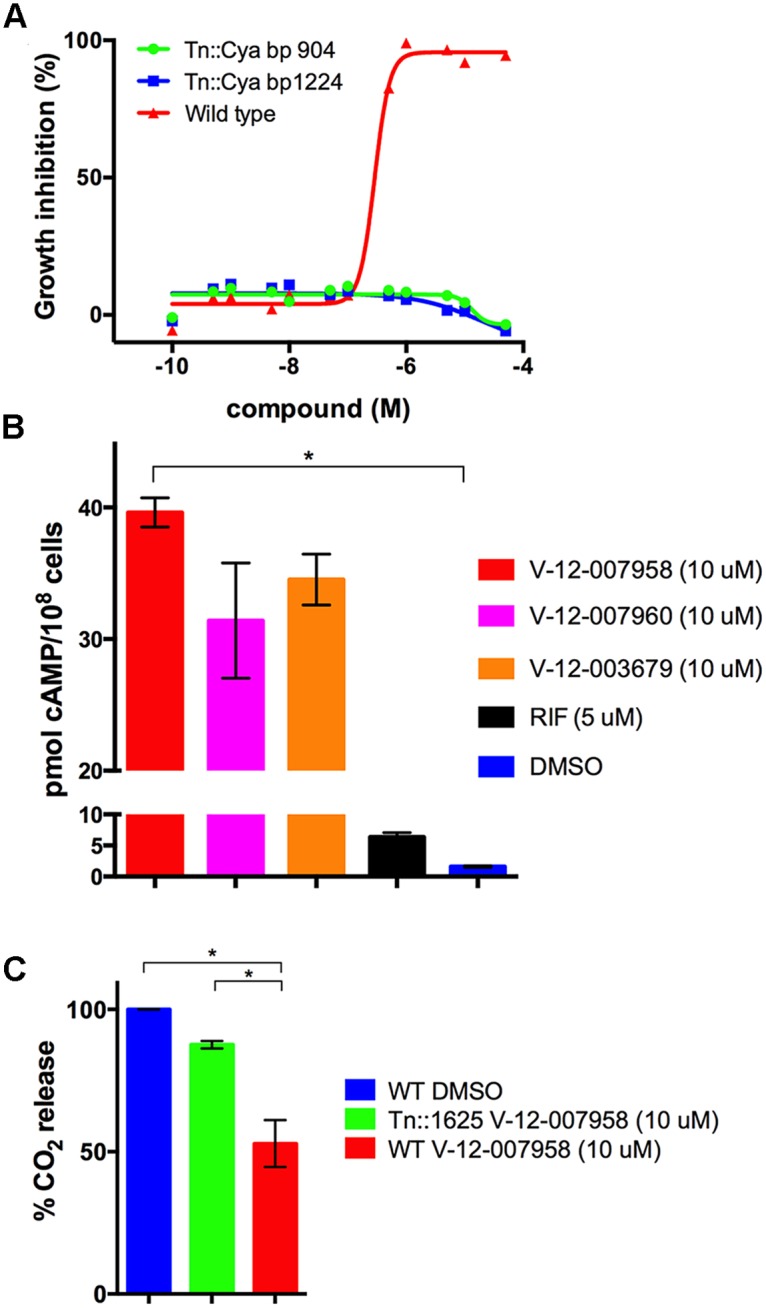
Inhibition of cholesterol utilization by the orphan inhibitor V-12–007958 is dependent on cAMP levels. **(A)** Transposon mutants with insertions in the gene *rv1625/cya* are resistant to V-12–007958 in 7H12 media containing cholesterol (100 μM). The transposon insertion sites in the *rv1625/cya* coding sequence are indicated. **(B)** The compounds V-12–003679, V-12–007958, and V-12–007960 all stimulate cAMP production in wild type Mtb grown in 7H12 media containing cholesterol (100 μM) and acetate (0.1%). **(C)** Cholesterol utilization is restored to levels similar to the uninhibited control in a transposon disrupted mutant with an insertion in *rv1625/cya*. Data are representative of at least two independent experiments, * = *P* < 0.05, and error bars represent s.d.

We therefore hypothesized that cAMP levels are perturbed by the orphan inhibitors, which negatively regulates cholesterol utilization. We first confirmed that two of the Tn::cya mutants were resistant to the compound V-12–007958 (Fig. 7A). We next quantified cAMP production by wild type Mtb following an 8-hour exposure to V-12–003679, V-12–007958, and V-12–007960 in 7H12 cholesterol media containing acetate. We observed that all three orphan compounds in this class significantly induced the production of cAMP (Fig. 7B). Lastly we confirmed that cholesterol utilization was no longer inhibited by V-12–007958 in the Tn::cya mutant in 7H12 cholesterol media containing acetate (Fig. 7C). Importantly, because acetate rescues growth inhibition by these compounds the reduction in the amount of ^14^CO_2_ and the production of cAMP in the presence of these compounds is not linked to a reduction in bacterial growth in 7H12 cholesterol media containing acetate. Although the precise molecular target for V-12–003679, V-12–007958, and V-12–007960 is unknown, this data suggests a role for high levels of cAMP in negatively regulating cholesterol utilization in Mtb.

## Discussion

One hurdle in TB drug discovery stems from a limited understanding of the growth conditions and physiological environments experienced by Mtb during infection. Historically, the conditions used to identify anti-Mtb compounds are artificial and are unlikely to resemble those conditions encountered by Mtb during infection [44]. It is known that Mtb experiences a variety of environmental stressors during the course of infection such as starvation, hypoxia and low pH [45]. We decided to directly incorporate the host macrophage into a drug screen to reproduce the most common niche exploited by Mtb in its host and to recapitulate at least some of the metabolic and physiological adaptations required for infection. We hypothesized that chemical interrogation of Mtb within the context of its host cell would reveal additional targets that would not be required in rich medium that can provide diverse metabolic escape routes that are absent within the macrophage environment. Our screening against Mtb in macrophages identified both conventional, universally-active compounds that functioned independently of the bacterial environment, and conditionally-active compounds that were active in macrophages or in medium with cholesterol as the limiting carbon source.

We were surprised to find that many of conditionally-active compounds required cholesterol for inhibitory activity in liquid culture and yet many of these compounds do not appear to target cholesterol utilization directly. Our interpretation is that cholesterol exerts a dominant influence on Mtb physiology in more ways than just being a substrate for energy production, perhaps by influencing carbon flux through central metabolic and biosynthetic pathways. More particularly, the unique mixture of central metabolites produced from cholesterol catabolism, such as acetyl-CoA, pyruvate, and propionyl-CoA, dictate that the bacteria make drastic metabolic rearrangements, which opens additional vulnerability to chemical intervention [27].

The inhibitors V-13–011503 and V-13–012725 are the first two known inhibitors of cholesterol catabolism in Mtb and they inhibit HsaAB, which is required for the NADH-dependent conversion of 3-HSA into 3,4-DHSA during degradation of the A/B rings of cholesterol. The HsaAB proteins function as an enzyme complex, it is currently unknown which protein is actually inhibited by these compounds. Our observation that chemical inhibition of HsaAB limits Mtb replication in macrophages is novel and is consistent with the prediction that these genes are required for growth in macrophages from previous transposon studies [17]. To our knowledge, survival studies with HsaAB mutants have not been reported in macrophages or *in vivo*. In addition to limiting Mtb replication in macrophages, compounds that target HsaAB may also inhibit extracellular Mtb replication in tissues where Mtb may potentially rely on an abundant pool of cholesterol within caseating granulomas [46]. Thus, efforts are currently underway to optimize the compounds, determine the precise molecular mechanism of HsaAB inhibition by these compounds, and to establish whether HsaAB inhibitors alone or in combination with frontline TB drugs enhance treatment outcomes in murine chemotherapy models *in vivo*.

Regulation of cholesterol utilization by cAMP has not been reported and this regulation may be governed at the transcriptional level or post-transcriptionally. It is known that cAMP levels can control Mtb metabolism by protein acetylation through the activity of the cAMP-dependent protein acetyltransferase Rv0998/PAT [47–49]. Importantly, several mycobacterial enzymes involved in lipid and propionate metabolism are acetylated by PAT [50,51] in a cAMP dependent manner, which may limit cholesterol utilization directly or act through feedback inhibition [51]. We did not identify transposon insertions in Rv0998/PAT but we cannot rule out protein acetylation as a mechanism involved here. The enzymes involved in cholesterol utilization in Mtb may be negatively regulated at the transcriptional level in the presence of high concentrations of cAMP. At present, the exact molecular target(s) for the orphan cholesterol utilization inhibitors remain to be determined. Our current model is that this family of compounds indirectly inhibit cholesterol utilization in Mtb by perturbing an unknown target or pathway that leads to the enhanced production of cAMP, which down regulates cholesterol utilization. More work is needed to define the precise role of cAMP in regulating cholesterol utilization. The ability of acetate to rescue growth inhibition by these compounds without impacting cholesterol utilization implies that these compounds ultimately starve Mtb by limiting entry of cholesterol-derived carbon into central metabolism.

Previous metabolic studies have shown that Mtb has the capacity to catabolize multiple carbon sources simultaneously [41], however the fates of carbons from the simple substrates glucose, glycerol and acetate are highly compartmentalized. The logical extension of this would predict that, under certain growth conditions, these substrates may not be interchangeable. Given the ability of acetate to rescue Mtb growth inhibition by these compounds in cholesterol medium it is puzzling that fatty acids or other nutrients fail to exhibit comparable activity in the macrophage. The results imply a partitioning of metabolism whereby, within in a macrophage, Mtb may preferentially utilize cholesterol for energy production while other nutrients such as carbohydrates or fatty acids may fulfill separate metabolic requirements [52–54]. Phenotypically, this appears as an unusual form of catabolite repression, however additional studies are needed to investigate this possibility directly.

The majority of compounds identified in this screen result in bacteriostatic phenotypes *in vitro*, which may limit their potential as lead compounds for drug development. However, the surprising diversity of targets, pathways, and mechanisms of action all linking back to cholesterol metabolism uncovers an extensive, and hitherto unappreciated chink in this bacterium’s armor.

## Methods

### Bacterial strains and media

*M*. *tuberculosis* CDC1551 and *M*. *tuberculosis* H37Rv Δicl1 [18] were utilized for all experiments. Bacteria were routinely grown at 37°C in 7H9 (broth) or 7H11 (agar) media supplemented containing 0.05% glycerol and OADC enrichment (0.5% bovine serum albumin fraction V, 0.2% glucose, 0.085% NaCl). Broth cultures also contained 0.05% tyloxapol. *E*. *coli* cultures were grown in LB medium. Antibiotics were added as described [53].

### HTS assay

Macrophages (J774 cells from American Type Culture Collection) were seeded into 384-well black clear bottom plates. *M*. *tuberculosis* CDC1551 expressing mCherry was grown to mid-log phase in Middlebrook 7H9 OADC washed, and syringed 6-times with 25G⅝ tuberculin syringe. The de-clumped bacteria were diluted into pre-warmed infection media (DMEM, 10% fetal calf serum, 2.0 mM L-glutamine, and 1.0 mM sodium pyruvate) and were used to infect cells at an MOI of 4:1. Bacteria were added to the screening plates with a Janus Ministation (Perkin Elmer). Compounds were added to the screening plates 1 hour after infection to a final concentration of 10 μM. Following a six day incubation period at 37°C and 6% CO_2_ Mtb mCherry fluorescence was quantified using an Envision Multilabel plate reader (Perkin Elmer). All screening plates contained negative (DMSO) and positive (10 μM rifampicin) controls. Percent inhibition for the experimental compounds was calculated using the formula, percent inhibition = 100x(DMSO signal—sample signal)/(DMSO signal—rifampicin signal). The Z′ factor, a measure of variability and reproducibility [21], was determined for each plate using the following formula: Z′ = 1-[3×(SD_rifampicin_+SD_DMSO_)/|M_rifampicin_-M_DMSO_|], where SD denotes the standard deviation and M denotes the mean for the samples and controls, respectively.

### Dose response assays against Mtb

To determine compound potency against Mtb in liquid culture an Alamar Blue reduction assay was used as described [22]. For inhibition assays conducted in Middlebrook 7H9 OADC the bacteria were cultured to mid-log phase (OD_600_ of 0.4) in 7H9 OADC and assayed in 96-well black clear bottom plates. Briefly, 1.0x10^6^ bacteria were added to each well containing 7H9 OADC and experimental compounds or controls to a final volume of 200 ul. For inhibition assays in media containing alternative carbon sources Mtb was first cultured to an (OD_600_ of 0.4) in 7H12 media (7H9 base, 0.1% casamino acids, 100 mM 2-morpholinoethanesulfonic acid pH 6.6) and 0.1% (wt/vol) acetate as the carbon source and 0.05% tyloxapol [25]. Cholesterol was added to the culture media at a final concentration of 100 μM as ethanol/tyloxapol micelles according to [53]. For the inhibition assay, bacteria were washed in PBS tyloxapol 0.05% twice and 1.0x10^6^ bacteria were added to 96-well microplates containing 7H12 media to a final volume of 200 μl containing the experimental compounds, controls, and supplemented with carbon substrates at 0.1% (wt/vol) unless otherwise noted. The microplates were incubated for 10 days in humidified, sealed plastic bags at 37°C. To quantify bacterial proliferation 40 ul of an Alamar Blue solution 50% was added to each well and the plates were re-incubated at 37°C for 16 hr. Alamar Blue reduction was quantified using an Envision Multilabel plate reader (Perkin Elmer) with λ_ex_ = 492 nm and λ_em_ = 595 nm.

To determine compound potency in the macrophages, J774 cells were infected and using the HTS protocol and exposed to a dilution series of the experimental compounds. All screening plates contained DMSO and 10 μM rifampicin control wells and percent inhibition for the experimental compounds was calculated. IC_50_ values were determined by fitting the percent inhibition dose response curves in Prism (GraphPad Software), using a sigmoidal variable slope fit with the maximum % activity and the minimum % activity fixed at 100% and 0%, respectively.

### Transcriptional profiling

Bacteria were grown in vented T-25 flasks as described above and treated with the experimental compounds at 10x IC_50_ concentration for 4 hours. Bacterial RNA was isolated, amplified, and labeled for microarray analysis. All the microarray hybridizations and data analyses were performed as described [7]. The entire microarray dataset is publically available on ArrayExpress database (www.ebi.ac.uk/arrayexpress/) accession number E-MTAB-3142.

### Radiorespirometry assays

Cholesterol utilization by *Mtb* was quantifying by monitoring the release of ^14^CO_2_ from [4–^14^C]-cholesterol by radiorespirometry. Briefly, Mtb cultures were grown in 5 ml 7H9 OADC or 7H12 medium supplemented with indicated carbon substrates in vented standing T-25 tissue culture flasks. Experimental compounds and 1.0 μCi of the radiolabel were added to the bacterial cultures at the same time. The culture flasks were placed air-tight vessel with an open vial containing 0.5 ml NaOH 1.0 M and sealed for incubation at 37°C. After 5 days, the NaOH vial was recovered, neutralized with 0.5 ml HCl 1.0 M, and the amount of base soluble Na_2_^14^CO_3_ was quantified by scintillation counting. Radioactivity signal was normalized to the relative levels of bacterial growth by determining the OD_600_ of the bacterial cultures at day 5.

### Recombinant 2-methylcitrate synthase assay

The full-length gene encoding PrpC/Rv1131 was cloned into pET23a (Novagen) creating a C-terminal fusion with a 6x-His tag. The recombinant PrpC was produced in *E*. *coli* BL21 (Novagen) following induction with isopropyl-thiogalactopyranoside 0.25 mM for 16 hours at 15°C. The His-tagged PrpC was purified from the *E*. *coli* lysates as described [55]. Methylcitrate synthase activity of the Mtb PrpC enzyme was monitored by detecting the release of CoASH, from propionyl-CoA during the condensation reaction with oxaloacetate, using 5,5′-dithiobis-2-nitrobenzoate as described [19]. The assays were conducted at 37°C in a 96-well black clear bottom plate containing 50 mM HEPES-NaOH pH 8.0, 0.1 M NaCl, 2 mM EDTA, 0.1 M DTNB, 0.14 mM propionyl-CoA, and 0.2 mM oxaloacetate. Recombinant PrpC (10 μg) was added and CoASH production was monitored spectrophotometrically at 412 nm using an Envision Multilabel plate reader. Background was subtracted to account for free thiol in the initial reaction mixture. The percent inhibition at each compound concentration was calculated using the equation %*I* = (1-*v*_*I/*_ *v*_*0*_)*100 where *v*_*I*_ and *v*_*0*_ are the rates observed in the presence and absence of inhibitor, respectively. The IC_50_ values were calculated by fitting to the inhibition data using nonlinear curve fitting.

### Analytical methods

Gas chromatography coupled mass spectrometry analyses of TMS-derivatized culture extracts were performed using an HP 6890 series GC system fitted with an HP 5973 mass-selective detector and a 30 m × 250 μm HP-5MS Agilent column. The operating conditions were: *T*_GC_ (injector), 280°C; *T*_MS_ (ion source), 230°C; oven time program *T*_0 min_, 104°C, *T*_2 min_, 104°C, *T*_14.4 min_, 290°C (heating rate 15°C·min^-1^), *T*_29.4 min_; 290°C.

### HsaAB inhibition kinetics

HsaA, HsaB, HsaC and HsaD were purified as described previously [29,31,32]. HsaB was reconstituted with FMN [29]. 3-Hydroxy-9,10-secoandrosta-1,3,5(10)-triene-9,17-dione (3-HSA), 3,4-dihydroxy-9,10-secoandrosta-1,3,5(10)-triene-9,17-dione (DHSA), and 4,5–9,10-diseco-3-hydroxy-5,9,17-tri-oxoandrosta-1(10),2-diene-4-oic acid (DSHA) were prepared using previously described biotransformations [29,31,32]. HsaC and HsaD assays were performed as described [31,32]. HsaAB activity was measured spectrophotometrically by following the hydroxylation of 3-HSA in a coupled assay with HsaC at 25 ± 0.5°C. Reactions were performed in 200 μl potassium phosphate (I = 0.1 M), pH 7.5 containing 2.5 μM HsaA, 1 μM HsaB, 1 μM HsaC, 400 μM NADH and 100 μM 3-HSA. Initial rates were determined over a 30 s interval. The reaction was monitored at 392 nm, the absorbance maximum of DSHA (ε_392_ = 3.8 mM^-1^ cm^-1^). Background ΔA_392_ was subtracted to account for uncoupled NADH consumption. Stock solutions were prepared fresh daily. The percent inhibition at each compound concentration was calculated using the equation %*I* = (1-*v*_*I/*_ *v*_*0*_)*100where *v*_*I*_ and *v*_*0*_ are the rates observed in the presence and absence of inhibitor, respectively. The IC_50_ values were calculated by fitting the equation %*I =* 100–100/(1+[I]/IC_50_) to the inhibition data using nonlinear curve fitting.

### Transposon mutant isolation

To isolate transposon mutants resistant to the inhibitor V-12–007958 a transposon library (~10^5^) in a wild type Mtb background was propagated for seven days in 7H12 media containing 100 μM cholesterol and 10 μM compound. Following the growth selection the bacteria were plated onto 7H11 OADC agar to isolate individual clones. Chromosomal DNA from the individual mutants was isolated and the transposon insertion sites were PCR amplified and sequenced according to [56].

### cAMP determination

Bacteria grown in 7H12 media containing cholesterol 100 μM cholesterol and 0.1% acetate were exposed to the experimental compounds for 8 hours. To determine intracellular levels of cAMP, cell suspensions containing 10^8^ cells were isolated by centrifugation. The bacterial pellet was suspended in 0.5 ml 0.1 M HCl and the cAMP containing lyaste was extracted by vigorous vortexing for 20 min. Bacterial debris was removed by centrifugation and the supernatants were used for cAMP estimation using a direct immunoassay kit (Enzo).

## Supporting Information

S1 Fig Whole cell HTS assay against Mtb in macrophages. **(A)** Dose response curve for the reference compound rifampicin tested against mCherry Mtb in the 384-well format infection assay. The data are representative of at least two independent experiments and the titration curve was fit using the percent inhibition values as described in methods. (**B**) Percent inhibition values from experimental compounds observed in a typical screening plate. This plate has a Z-factor = 0.75 the green line denotes mean percent inhibition and the red line denotes 3 s.d. from the mean.
(TIFF)

S2 Fig Chemical rescue of ΔIcl1 Mtb in 7H9 OADC supplemented cholesterol or propionate. Mtb ΔIcl1 mCherry signal was quantified following 12 days incubation in 7H9 OADC supplemented with 100 μM cholesterol or 100 μM propionate. Experimental compounds were tested at a concentration was 10 μM and carbon supplements are indicated in parentheses. The bacterial mCherry signal is expressed on a Log 10 scale and the data represents a single point read from a screening plate.
(TIFF)

S3 Fig GC-MS analyses of culture extracts from Mtb cells treated with V-13–011503 or V-13–012725 detects 3-HAS accumulation. **(A)** The retention time and MS spectra of Peak 1 (*t*_R_ = 14.9 min) corresponds to 3-HSA. The retention time and MS spectra of Peak 2 (*t*_R_ = 18.9 min) corresponds to that of 3-HSBNC. The retention time and MS spectra of Peak 3 (*t*_R_ = 19.7 min) is consistent with that of an unsaturated 3-HSBNC although the position of the double bond could not be determined due to the low yield of metabolite. These three peaks are absent from DMSO-treated Mtb extracts. **(B)** Cholesterol catabolic pathway, lower route indicates the successive actions of HsaAB, HsaC and HsaD on sterol rings A and B. The R at C17 of cholesterol indicates that the extent of side chain can vary. The respective substrates of HsaAB, HsaC and HsaD are 3-HSA, 3,4-DHSA and DSHA. The C4 of cholesterol is indicated, and colored red.
(TIFF)

S4 Fig Structures of the three orphan cholesterol utilization inhibitors. (TIFF)

S1 Table Genes up- and down-regulated in response to the orphan cholesterol utilization inhibitors. (XLSX)
