# Supplementary figures and images for: Novel Inhibitors of Cholesterol Degradation in Mycobacterium tuberculosis Reveal How the Bacterium’s Metabolism Is Constrained by the Intracellular Environment
Source: PLoS Pathog. 2015 Feb 12;11(2):e1004679. doi: 10.1371/journal.ppat.1004679 (PMC4335503; doi:10.1371/journal.ppat.1004679)

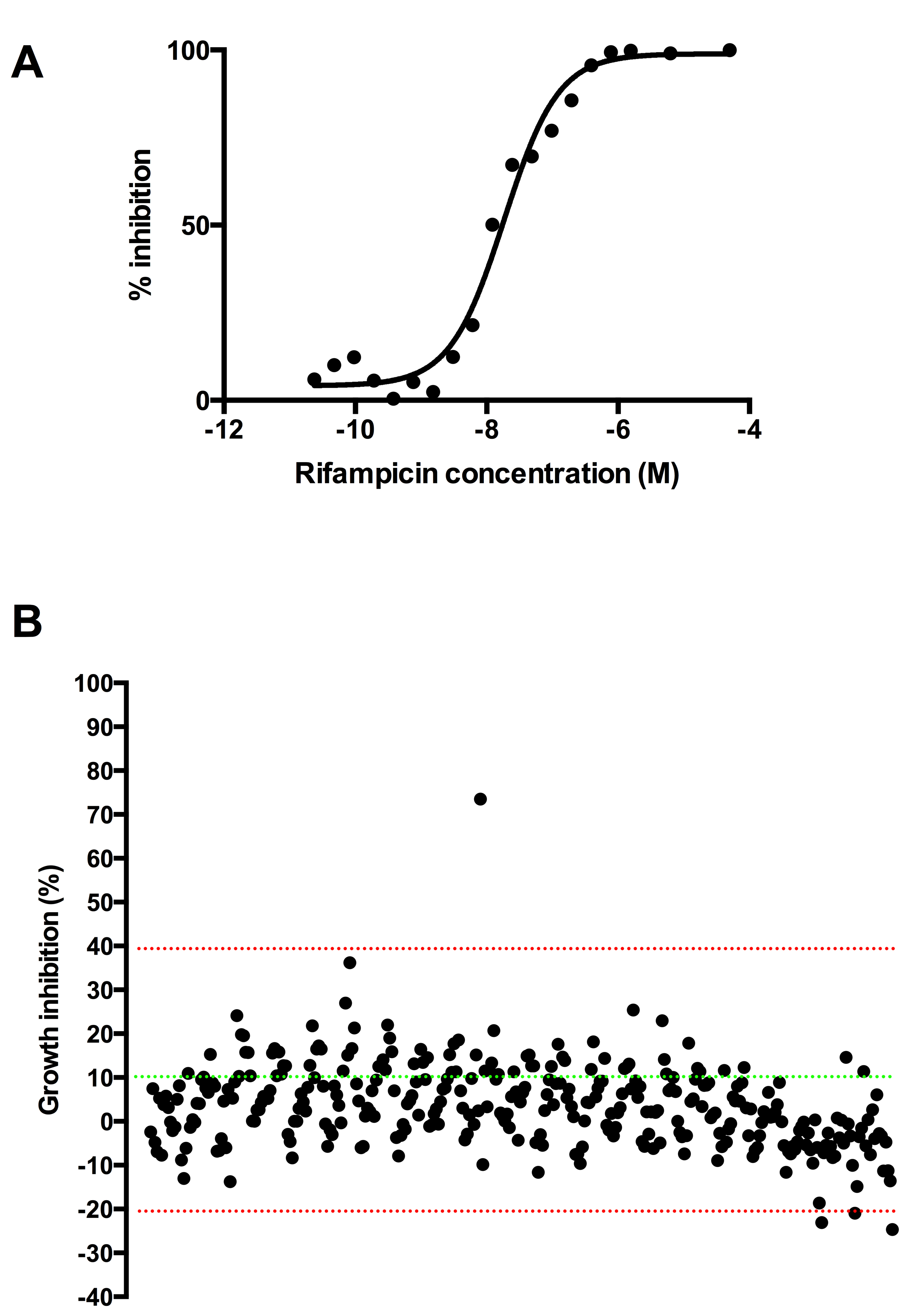

Supplement: S1 Fig — (A) Dose response curve for the reference compound rifampicin tested against mCherry Mtb in the 384-well format infection assay. The data are representative of at least two independent experiments and the titration curve was fit using the percent inhibition values as described in methods. (B) Percent inhibition values from experimental compounds observed in a typical screening plate. This plate has a Z-factor = 0.75 the green line denotes mean percent inhibition and the red line denotes 3 s.d. from the mean. (TIFF) [file ppat.1004679.s001.tiff]

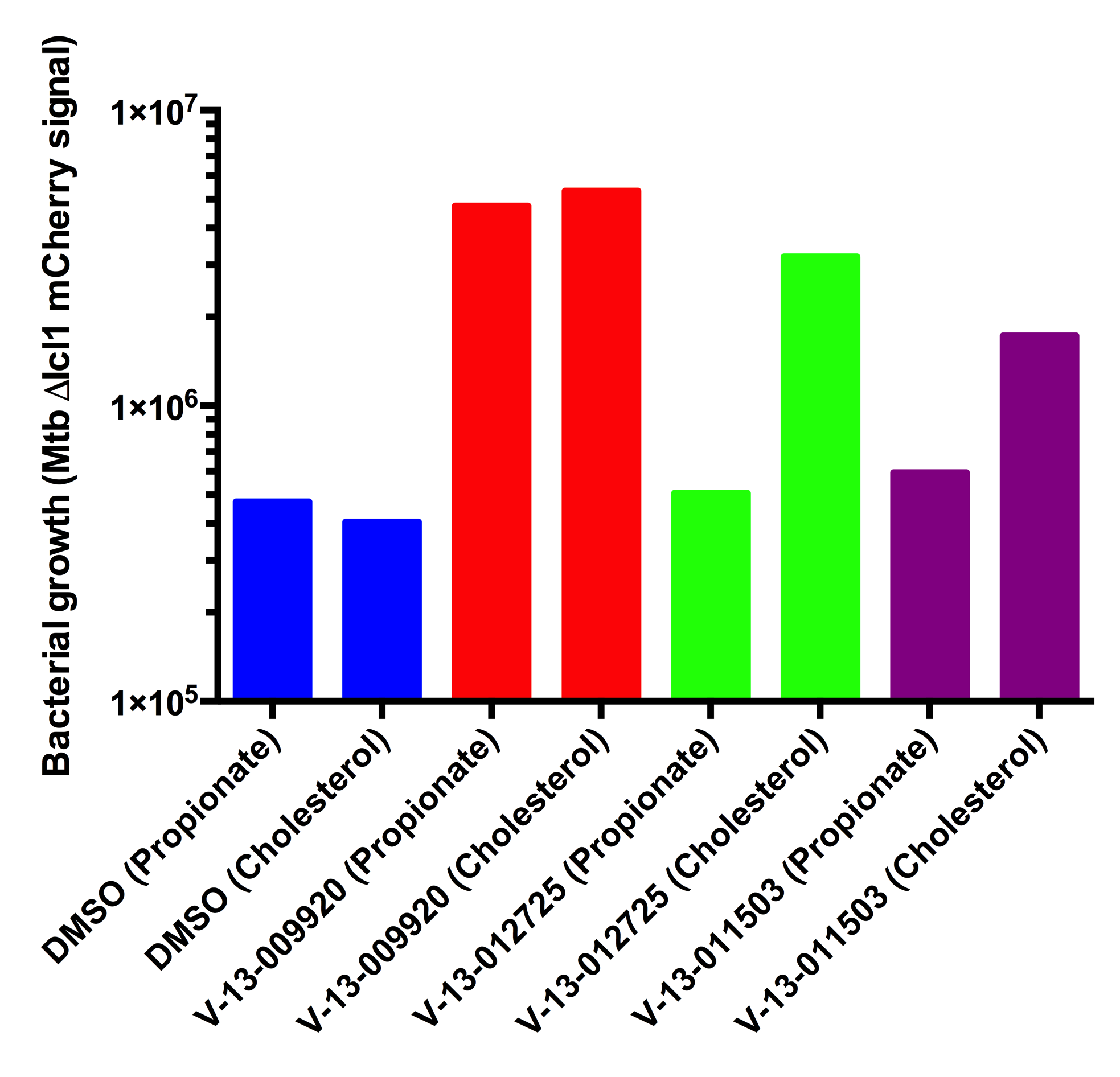

Supplement: S2 Fig — Mtb ΔIcl1 mCherry signal was quantified following 12 days incubation in 7H9 OADC supplemented with 100 μM cholesterol or 100 μM propionate. Experimental compounds were tested at a concentration was 10 μM and carbon supplements are indicated in parentheses. The bacterial mCherry signal is expressed on a Log 10 scale and the data represents a single point read from a screening plate. (TIFF) [file ppat.1004679.s002.tiff]

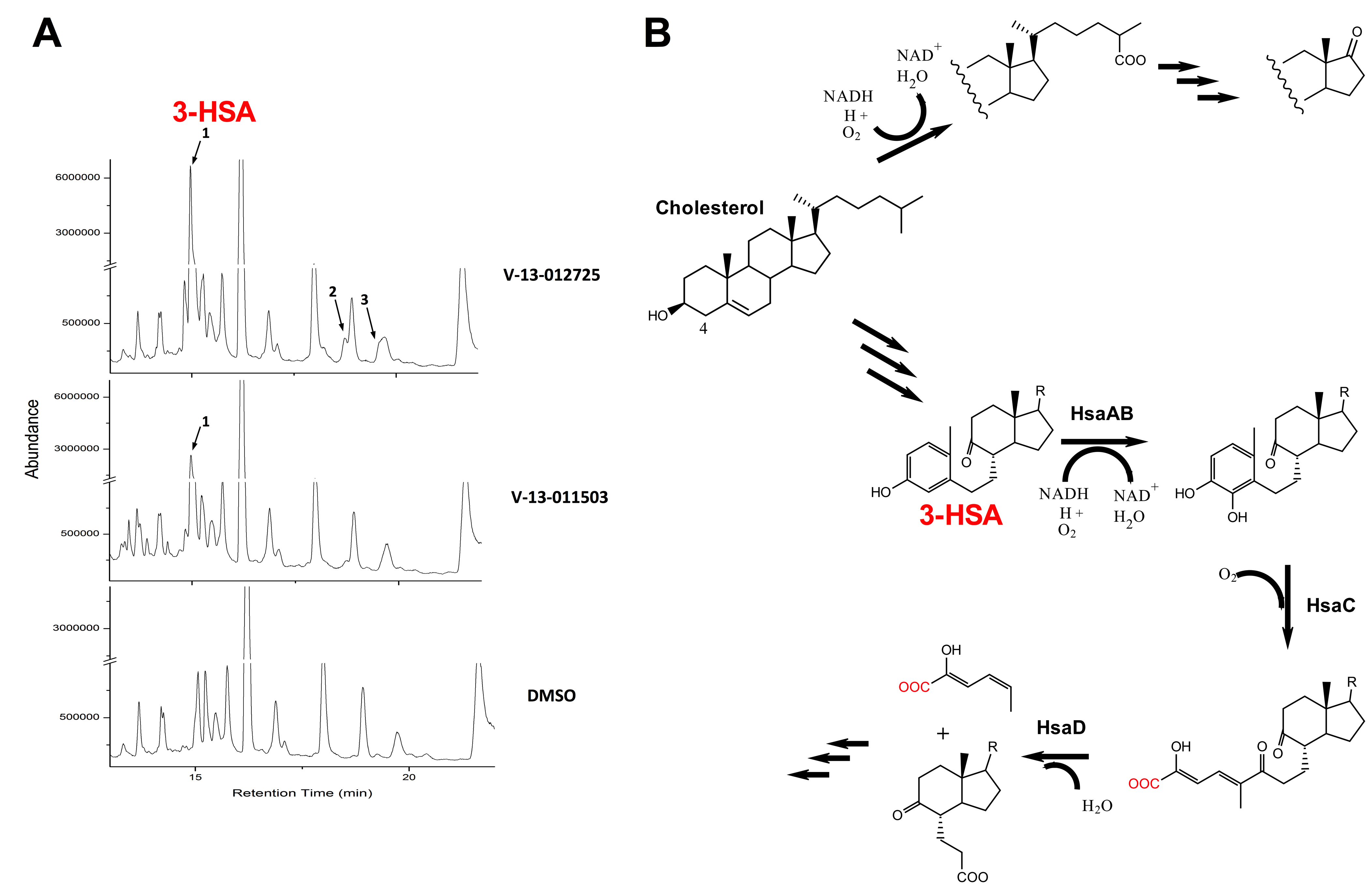

Supplement: S3 Fig — (A) The retention time and MS spectra of Peak 1 (t R = 14.9 min) corresponds to 3-HSA. The retention time and MS spectra of Peak 2 (t R = 18.9 min) corresponds to that of 3-HSBNC. The retention time and MS spectra of Peak 3 (t R = 19.7 min) is consistent with that of an unsaturated 3-HSBNC although the position of the double bond could not be determined due to the low yield of metabolite. These three peaks are absent from DMSO-treated Mtb extracts. (B) Cholesterol catabolic pathway, lower route indicates the successive actions of HsaAB, HsaC and HsaD on sterol rings A and B. The R at C17 of cholesterol indicates that the extent of side chain can vary. The respective substrates of HsaAB, HsaC and HsaD are 3-HSA, 3,4-DHSA and DSHA. The C4 of cholesterol is indicated, and colored red. (TIFF) [file ppat.1004679.s003.tiff]

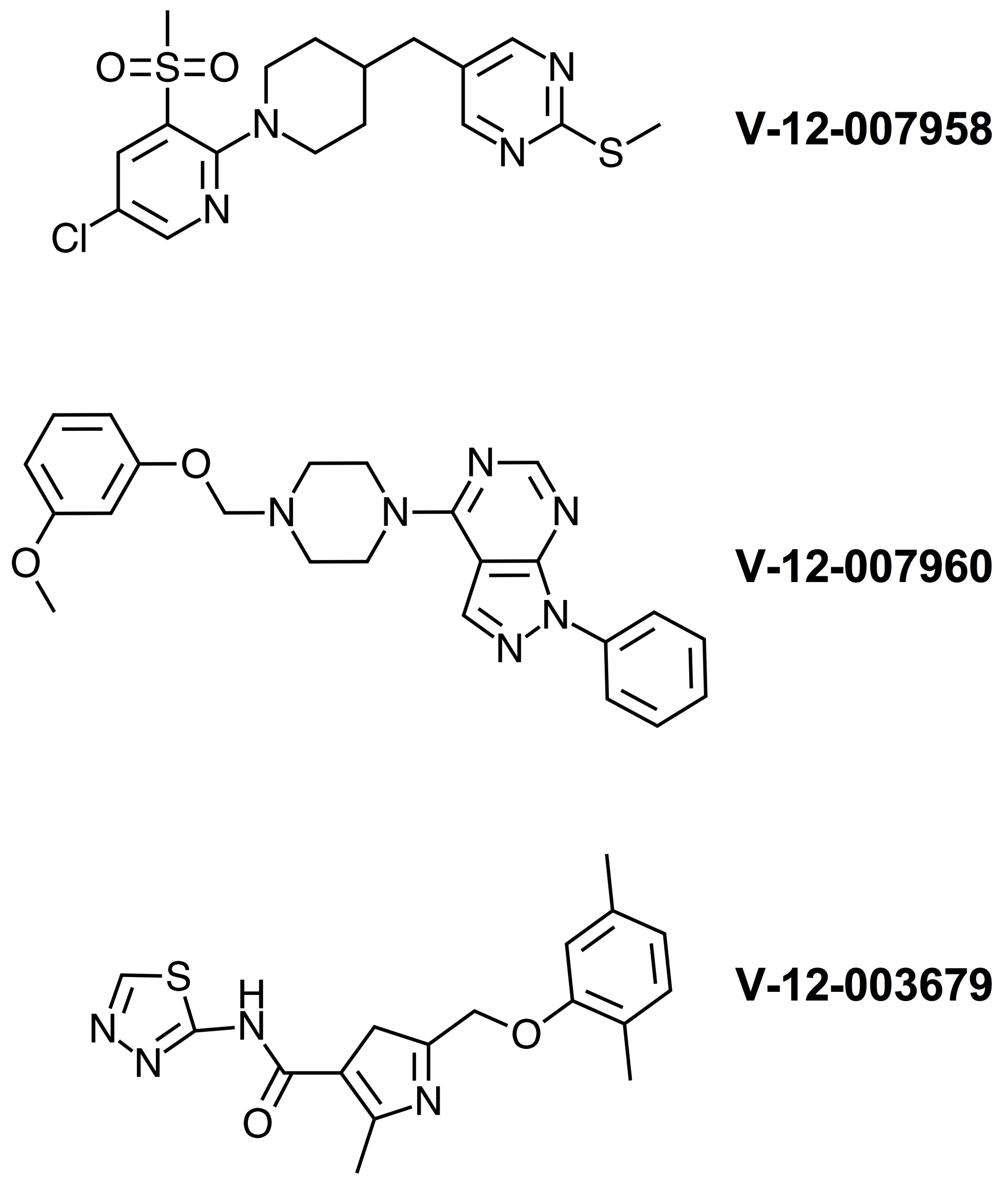

Supplement: S4 Fig — (TIFF) [file ppat.1004679.s004.tiff]
